# Repurposed FDA-approved drugs targeting genes influencing aging can extend lifespan and healthspan in rotifers

**DOI:** 10.1007/s10522-018-9745-9

**Published:** 2018-01-16

**Authors:** Terry W. Snell, Rachel K. Johnston, Amelia B. Matthews, Hongyi Zhou, Mu Gao, Jeffrey Skolnick

**Affiliations:** School of Biological Sciences, Georgia Institute of Technology, Atlanta, GA 30332-0230 USA

**Keywords:** Aging, Re-purposing drugs, Rotifera, Lifespan, Healthspan, Aging genes

## Abstract

Pharmaceutical interventions can slow aging in animals, and have advantages because their dose can be tightly regulated and the timing of the intervention can be closely controlled. They also may complement environmental interventions like caloric restriction by acting additively. A fertile source for therapies slowing aging is FDA approved drugs whose safety has been investigated. Because drugs bind to several protein targets, they cause multiple effects, many of which have not been characterized. It is possible that some of the side effects of drugs prescribed for one therapy may have benefits in retarding aging. We used computationally guided drug screening for prioritizing drug targets to produce a short list of candidate compounds for in vivo testing. We applied the virtual ligand screening approach FINDSITE^comb^ for screening potential anti-aging protein targets against FDA approved drugs listed in DrugBank. A short list of 31 promising compounds was screened using a multi-tiered approach with rotifers as an animal model of aging. Primary and secondary survival screens and cohort life table experiments identified four drugs capable of extending rotifer lifespan by 8–42%. Exposures to 1 µM erythromycin, 5 µM carglumic acid, 3 µM capecitabine, and 1 µM ivermectin, extended rotifer lifespan without significant effect on reproduction. Some drugs also extended healthspan, as estimated by mitochondria activity and mobility (swimming speed). Our most promising result is that rotifer lifespan was extended by 7–8.9% even when treatment was started in middle age.

## Introduction

Because of the infirmities associated with human aging, there continues to be great interest in interventions that can mitigate the process. Of the three approaches, genetic manipulation continues to make important contributions to the scientific understanding of the mechanisms of aging, whereas environmental and pharmacological interventions offer the most promise for practical benefits. Little is known about how pharmaceutical interventions slow aging in animals, but they have advantages because their dose can be tightly regulated and the timing of the intervention can be closely controlled. Pharmacological interventions also may complement environmental interventions like caloric restriction by acting additively.

A fertile field to search for therapies that can slow aging is the pool of FDA approved drugs whose safety has been thoroughly investigated (Armanios et al. 2015). All drugs bind to several protein targets causing multiple effects (Zhou et al. 2015), many of which have not been biologically characterized. It is possible that some of the side effects of drugs prescribed for one type of therapy may have benefits in slowing aging. The field of re-purposing approved drugs for other therapies is rapidly growing (Ashburn and Thor 2004; Pantziarka et al. 2014).

Some metabolic pathways are known to be key in aging like insulin/IGF-1 signaling involved in nutrient sensing and metabolic regulation. For a variety of reasons, drug development has targeted many proteins in this pathway as therapy for a diversity of diseases. However, there has been little systematic drug development aimed at aging therapy. Moreover, there could be other metabolic pathways not yet associated with aging that respond to pharmacological intervention with existing approved drugs.

With more than 5000 drugs in commercial use, it would be quite difficult to test all of these experimentally for aging benefits using in vivo animal models. An alternative is to use computationally guided drug screening for prioritizing drug targets (Snell et al. 2016; Calvert et al. 2016; Ziehm et al. 2017). This would produce a short list of candidate compounds that could then be subjected to the power of in vivo testing with animals. This is one of the most promising strategies for identifying pharmacological interventions that can safely slow aging and extend human lifespan and healthspan.

In this paper we have emphasized testing for effects vs biochemical mechanisms. Our priority has been to screen as many drugs as quickly as possible for major beneficial effects slowing aging. Once a small pool of such compounds has been identified, then a concerted effort can be mounted to understand the biochemical mechanisms underlying the therapeutic effects.

We apply the virtual ligand screening approach FINDSITE^comb^ (Zhou and Skolnick 2013) for screening potential aging-related protein targets against FDA approved drugs listed in DrugBank (Wishart et al. 2006) to identify drugs with the potential to slow aging. FINDSITE^comb^ has advantages over traditional ligand based approaches in that it does not require a known set of ligands for the target. It is also advantageous to traditional structure-based docking methods because it does not require high-resolution target structures and screens a compound library much faster; and more importantly has much better enrichment factor. FINDSITE^comb^ generates a short list of FDA drugs that potentially bind to aging-related protein targets of an animal model. This short list of promising compounds was screened using a multi-tiered approach with rotifers as an animal model of aging. Rotifers have several advantages as experimental animal models for investigating the biology of aging (Snell 2014; Snell et al. 2014a, b). Among these are a short life cycle so that cohort life table experiments can be completed within 3 weeks, the ability to clone females via parthenogenetic reproduction, the possibility of performing experimental evolution in chemostats, and the identification in rotifer transcriptomes of many genes implicated in aging and their homology to similar genes in mammals.

In this paper, we screened a short list of drug candidates with the potential to extend lifespan using in vivo animal experiments with primary and secondary survival screens followed by cohort life tables. We also screened drugs for their ability to extend healthspan by using proxies for mitochondrial activity and swimming speed (mobility) during the aging process. We found a few drugs capable of extending both lifespan and healthspan and then tested them for additive or synergistic effects in combined exposures. In addition to the beneficial effects of life-long therapy of some drugs, we demonstrated retarded aging effects in a few cases even when drug therapy was initiated in midlife, a phenomenon that many consider the holy grail of aging therapy.

## Materials and methods

### Computational screening of putative aging proteins and FDA-approved drugs

Protein targets for drug binding to *Brachionus manjavacas* were identified in a multistep process (Table 1) beginning with identifying putative aging genes from the GenAge database (http://genomics.senescence.info/genes/stats.php). We used data from the October 8, 2015 build, which identified 2054 putative aging genes from 9 model organisms, including *Saccharomyces cerevisiae, Caenorrhabditis elegans, Drosophila melanogaster,* and *Mus musculus*. The genes were ranked by their effect on maximum average lifespan increase, so we chose those in these four model organisms whose knock down produced a > 20% increase in average lifespan. From this pool of protein candidates, we identified 94 proteins with > 40% amino acid sequence similarity to *Adineta vaga* genes, the only rotifer for which a whole genome analysis is currently available (Flot et al. 2013).

**Table 1:**
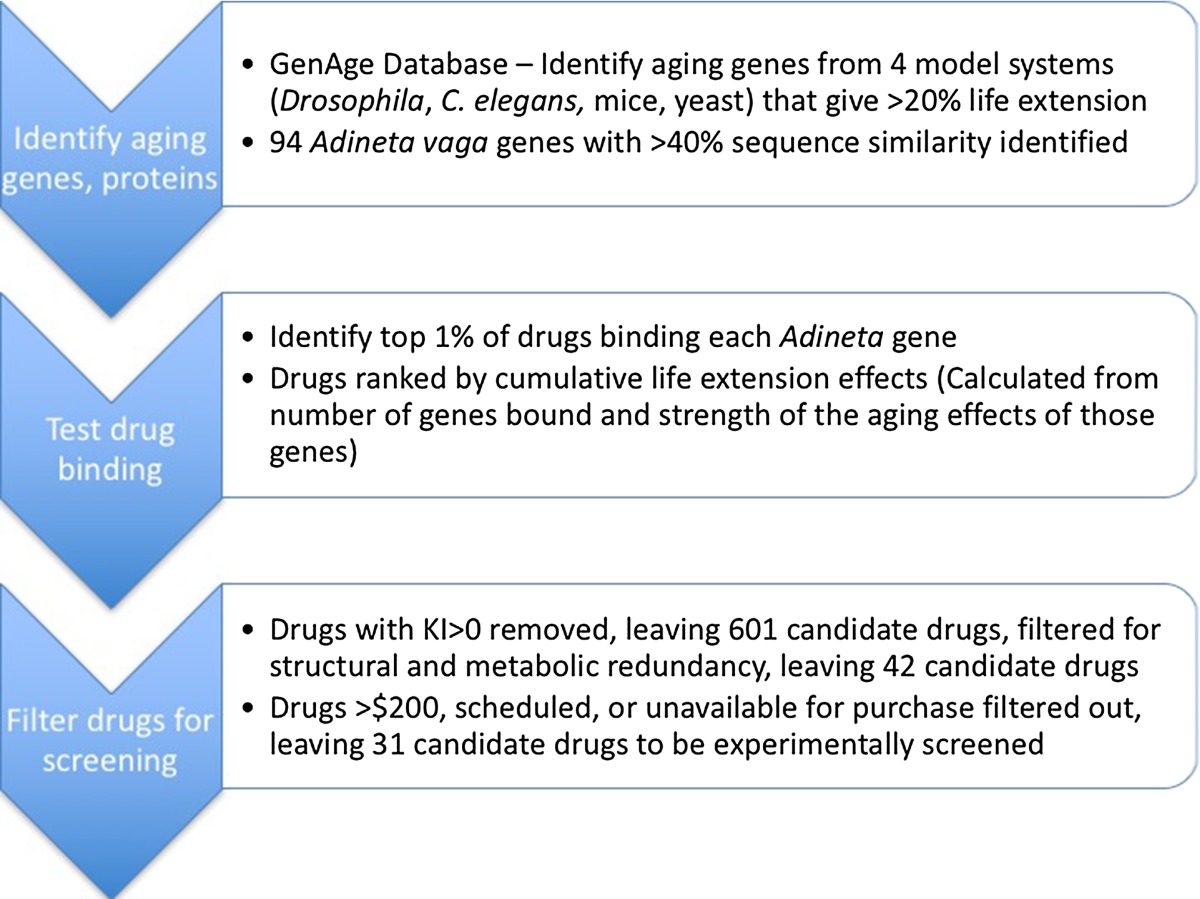
Flow chart of drug screening

Subsequently, FINDSITE^comb^ was applied to screen the 94 proteins against all DrugBank drugs (including FDA approved & experimental drugs) plus the ZINC8 molecules clustered at Tanimoto Coefficient (TC) 0.8 as background (Irwin and Shoichet 2005). FINDSITE^comb^ takes the protein amino acid sequence as input and builds a structural model using a threading approach. The pockets of each target protein were detected in their models, and subsequently were compared to the ligand-binding pockets found in PDB structures (Bernstein et al. 1977) and to the pockets of the structural models of the proteins from the ChEMBL (Gaulton et al. 2012) and DrugBank (Wishart et al. 2006) libraries. Pockets with the most significant similarity to the target pockets were selected and their corresponding binding ligands were used as template ligands. These template ligands were then utilized as seed ligands for virtual screening against the compound library with a fingerprint comparison method (Nikolova and Jaworska 2003).

The compounds were ranked in a list by their similarity score to the seed ligands. FDA approved drugs within top 1% of the screening list for each *Adineta* gene product are identified as a potential binder of the protein. We then ranked each drug according to its cumulative aging effects as the summed strength of the life extension effects of those proteins predicted to bind to the drug (Table 2). Although FDA approved drugs are mostly safe, some of them still have serious side effects and can be filtered with a killing index (KI) that are associated with severe side effects such has heart attack, cancer and death (Zhou et al. 2015). Drugs with KI > 0 were removed, leaving 601 candidate drugs. Top 100 drugs were filtered for structural and experimental redundancy by clustering them at TC = 0.8, leaving 42 candidate drugs. Drugs costing more than $200, scheduled, or unavailable for purchase were also filtered out, leaving 31 candidate drugs to be experimentally screened using the rotifer model.

**Table 2:**
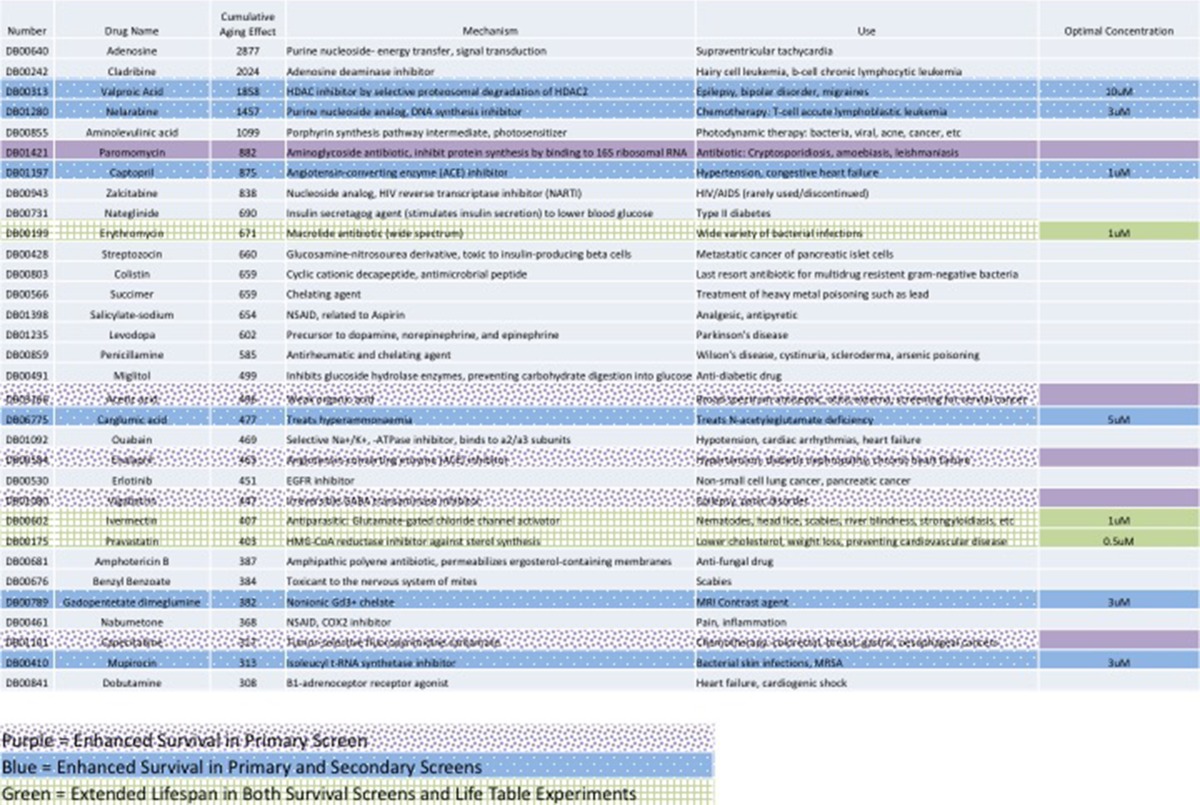
Summary of drugs screened

Number refers to a drug’s DrugBank number. Cumulative aging effect is the summed strength of the life extension effects from the GenAge database. This provides an estimate of the “maximum average lifespan change” for each gene as  % effect on lifespan. For example, the *C. elegans* let-363 gene extends worm lifespan by about 150%. A rotifer target mapped to this gene will receive an aging effect of 150. The Cumulative aging effect for a drug is the sum of all of these effects for all putative rotifer protein targets. Optimal concentration is the experimentally determined drug concentration that produced significantly longer lifespan or healthspan. Purple highlighting—enhanced survival in primary screen, black highlighting—enhanced survival in secondary screen, green highlighting—enhanced survival in primary and secondary screens and life table experiments

### Experimental design and treatments

Experiments were performed with the rotifer species *Brachionus manjavacas* (Russian strain), which was cultivated in 15ppt salinity, and fed the green alga *Tetraselmis suecica*, as described in Snell et al. (2016). All treatments were applied by dissolving the drugs in water and adding them to the rotifer medium. When DMSO was used as a solvent to assist drug solubility, the controls also contained an identical concentration of DMSO. DMSO concentration was kept below 0.2% for all treatments, a concentration shown in many trials to have no effect on rotifer lifespan or reproduction.

Full cohort life table and survival screen experiments were conducted following the methods detailed in Snell et al. (2016). Life table experiments were performed with a cohort of 120 neonate rotifers in each treatment, 5 per well in a 24-well plate. Each well contained 6 × 10^5^ cells/mL *T. suecica* in 15 ppt artificial seawater (ASW), drug treatments, and 20 μM 5-fluoro-2-deoxyuridine (5-FU or FDU) which prevents hatching of asexual eggs (Snell et al. 2012). Plates were incubated at 22 °C and scored daily for mortality. In experiments where drug treatments were delayed, the rotifers were transferred to new plates with fresh medium containing the appropriate drug treatments on Day 9.

Survival screens were conducted with a cohort of 84 rotifer neonates per treatment, 7 per well in 12 wells of a 24-well plate. These rotifers were maintained identically as in the life table experiments. Five μL 5-FU (1 mg/mL) was added to each well on days 2, 4, and 6 to prevent egg hatching, and *T. suecica* food was replenished on day 6. These plates were incubated at 28 °C, and the number of live animals was counted on day 10. Survival was scored as the average percent surviving in each well on day 10. All drugs were tested in primary survival screens at both 1 and 5 μM concentrations. Drugs that enhanced survival in primary screens were tested in a secondary survival screen at three concentrations (ranging from 0.5 to 10 μM) chosen based on the results of the primary screen.

### Assessment of rotifer healthspan

Drugs were tested for their ability to extend rotifer healthspan by analyzing reproductive rates, mitochondrial activity, and swimming speed using the methods described in Snell et al. (2016).

Reproductive life table experiments were conducted as described above with the exception of using a cohort of 24 rotifer hatchlings per treatment, one per well in a 24-well plate, and no 5-FU in the medium to allow normal egg hatching. Offspring were counted and removed each day, and intrinsic population growth rate (r) was calculated for each treatment.

Mitochondrial activity was estimated using MitoTracker^®^ Red (Invitrogen). Rotifers were incubated with drug treatments, the alga *T. suecica*, 15 ppt ASW, and 20 μM 5-FU at 22 °C for 4 days. They were then rinsed and incubated with 5 μM MitoTracker^®^ Red in the dark for 30 min. After rinsing again, rotifers were anesthetized with club soda, fixed with formalin, and imaged on a Zeiss Imager Z1 microscope. Images were taken at ×200 magnification with an Alexa 568 nm filter, and average pixel intensity was measured using ImageJ. In experiments where drug treatments were delayed, rotifers were transferred into the drug treatments on day 9 and stained and imaged on days 10, 12, 14 and 16. MitoTracker requires active mitochondria to yield a fluorescent product; mitochondria in dead rotifers do not fluoresce (Snell et al. 2014b). However, MitoTracker should only be regarded as imprecise measure of mitochondrial activity as compared to other measures of mitochondrial metabolism (Brand and Nicholls 2011).

To measure swimming speed, rotifers were first incubated with drug treatments, *T. suecica*, 15ppt ASW, and 20 μM 5-FU at 22 °C for 10 days. On day 10, 15 rotifers from each treatment were transferred to a microscope slide in 12 μL ASW. Video of swimming behavior was recorded for 30 s using a PixeLink camera on a stereomicroscope at ×10 magnification. Swimming speed was then calculated for 10 rotifers from each treatment using the Tracker Video Analysis and Modeling Tool program (http://physlets.org/tracker/). In experiments where drug treatments were delayed, rotifers were transferred into the drug treatments on day 9 and videos were taken and analyzed on days 10, 12, 14, and 16.

### Statistics

Survival screens and healthspan assessments (average reproduction per female, swimming speed, MitoTracker^®^ Red fluorescence) were analyzed using an ANOVA with Dunnett’s test comparing treatments to control. Life table experiments were analyzed by using the JMP Pro 12 (SAS Institute) reliability and survival analysis with Wilcoxon’s test to compare survival curves.

## Results

The flow diagram in Table 1 illustrates the rationale for selecting drugs to test for their effects on aging. Our models identified 31 drugs with favorable binding patterns to be experimentally screened for lifespan extension. Most of these drugs had never been implicated in any effects on aging. A list of these drugs, their mechanisms of action, medical use, and therapeutic dose is shown in Table 2.

**Fig. 1:**
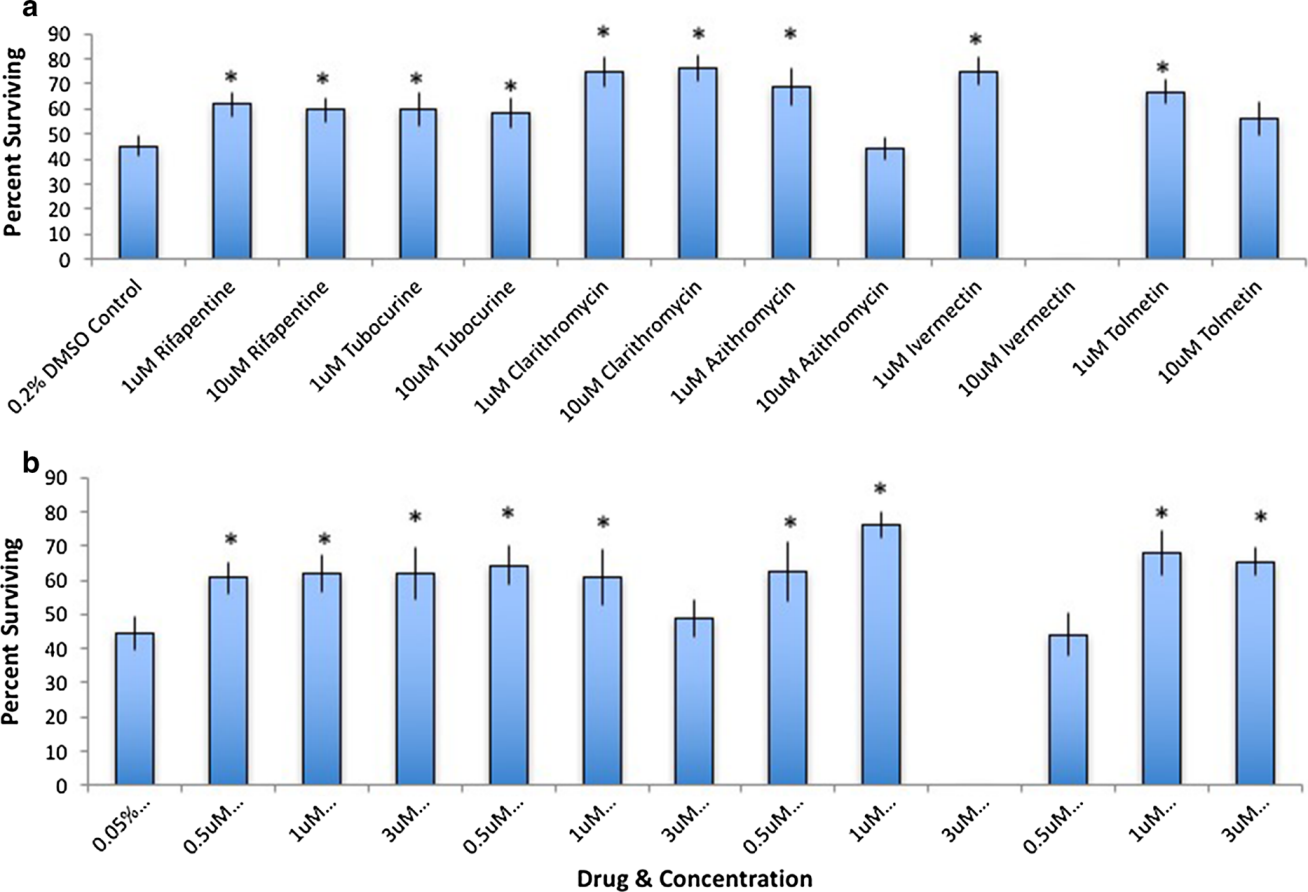
**a**, **b** 10 day primary and secondary survival screens for 6 drugs binding to aging pathway proteins. Asterisks indicate treatments where survival is significantly higher than control (P < 0.05)

Our experimental design called for a series of screens for drug effects on rotifer survival. Because rotifers are aquatic animals, all exposures were with drugs dissolved in water. Survival after 10 days of continuous drug exposure was compared to either a control of the dilution water or a solvent control that consisted of the dilution water plus 0.2% DMSO (if drug solubility required a carrier). An example of a primary drug screen can be seen in Fig. 1a. Rotifer survival after 10 days exposure to six drugs at 1 and 10 µM concentrations was compared to a control containing DMSO. Asterisks above the column indicate significantly better survival than control by ANOVA and Dunnett’s test. For example, survival was improved by 67% over control by exposure to 1 µM of the drugs clarithromycin and ivermectin. In contrast, exposure to 10 µM ivermectin killed all rotifers in the 10 day exposure. All 31 drugs screened were subjected to a primary screen. Drugs yielding significant lifespan extension in at least one concentration were subjected to a secondary screen (Fig. 1b). A secondary screen was a similar experiment as a primary screen, but with different drug concentrations. For example, ivermectin was tested at 1 and 3 µM. At 1 µM exposure lifespan was once again extended 71% over control, but all rotifers died when exposed to 3 µM ivermectin.

**Fig. 2:**
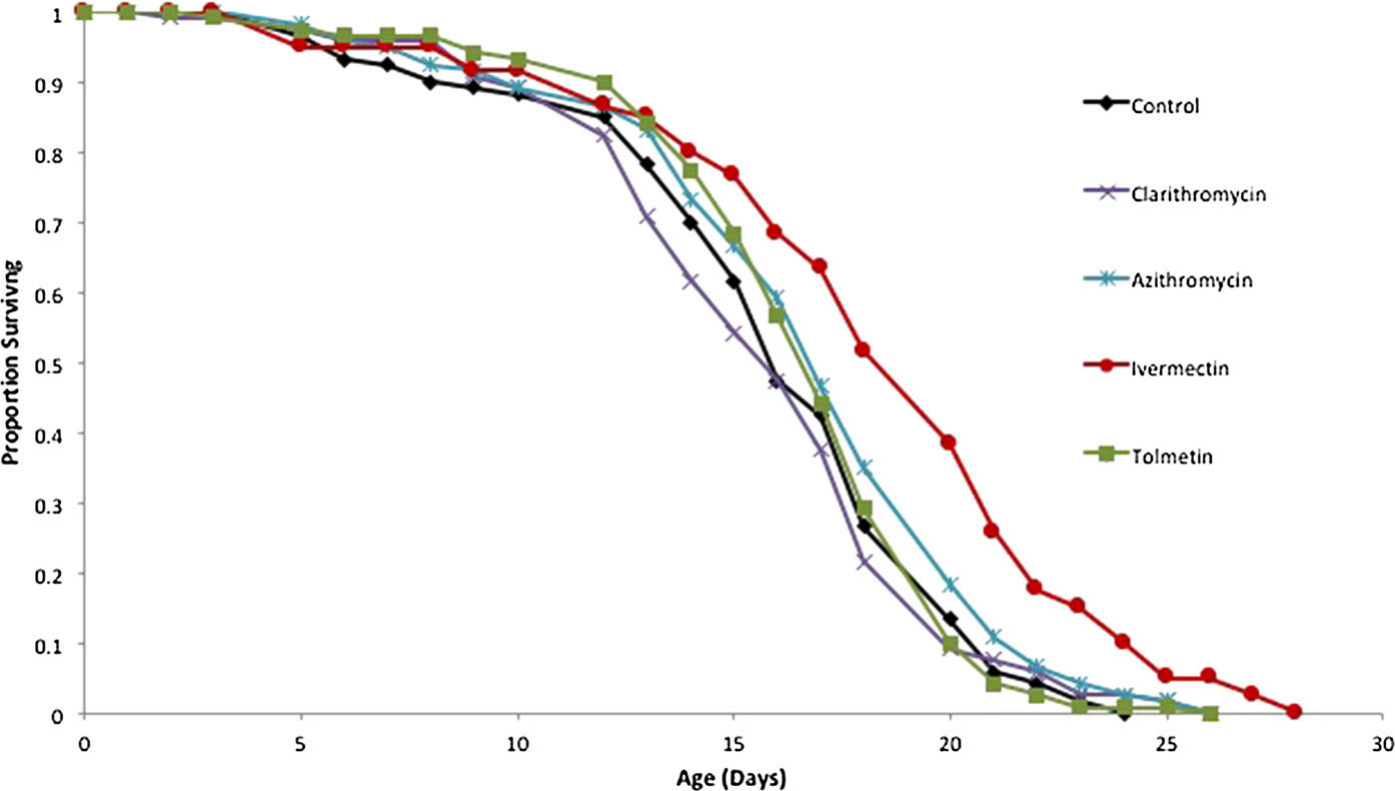
Cohort life table with 120 rotifers per treatment of 4 drugs binding to aging pathway proteins. Mean lifespan in the ivermectin treatment is significantly longer than control (P = 0.0049)

**Fig. 3:**
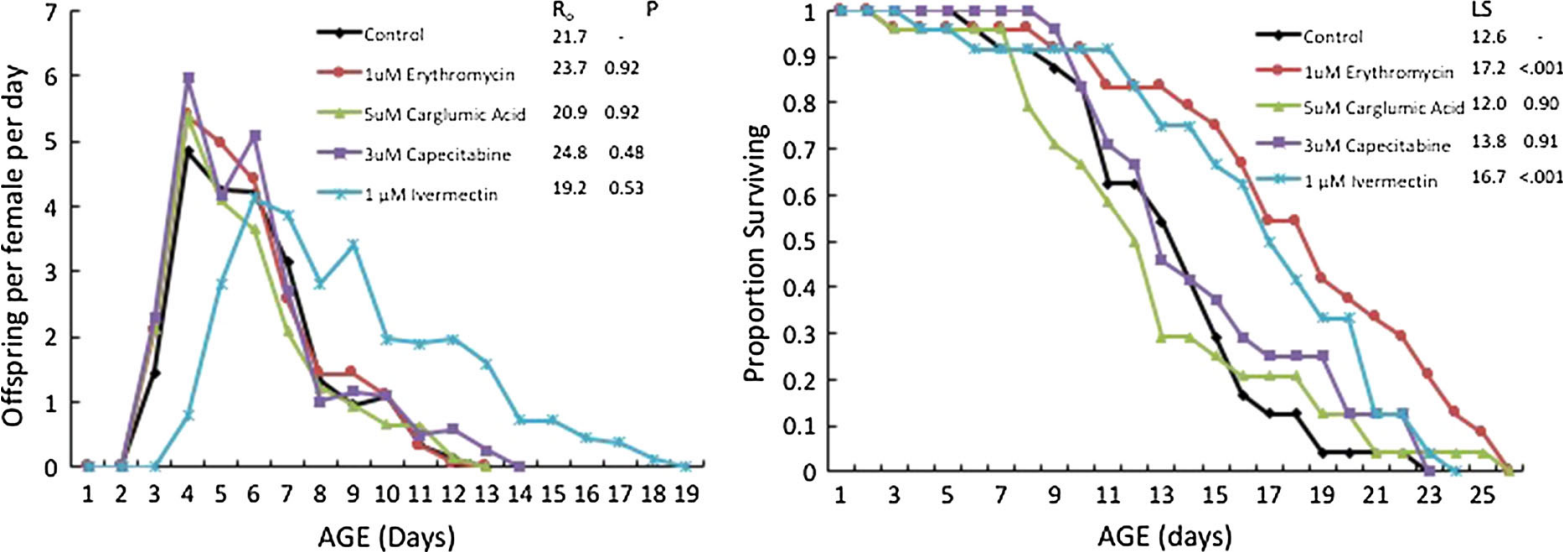
Reproductive life table with 24 rotifers per treatment of 4 drugs binding to aging pathway proteins. *Ro* net fecundity of females over their reproductive lifetimes, *P* probability of significant difference from control by one-way ANOVA and Dunnet’s test, *LS* mean lifespan (days), *P* probability of significant difference in lifespan from control by Wilcoxson test

Drugs giving positive results in primary and secondary screens were then subjected to a full life table analysis where test animals were exposed to a drug from birth to their death about 3 weeks later (Fig. 2). Once again, 1 µM ivermectin produced the best results in this experiment comparing four drugs, with a 8% longer mean lifespan, 13% longer median lifespan, and a 19% longer maximum lifespan (age of 95% mortality) than control. Primary and secondary screens as well as life table experiments were performed with the drug 5-FU in the media to prevent hatching of eggs. This facilitates the performance of these experiments by eliminating any confusion among maternal and F1 females. However, a reproductive life table also needs be performed to check that candidate drugs do not significantly inhibit reproduction. An example of a reproductive life table experiment is shown in Fig. 3. It can be seen that among 1 µM erythromycin, 5 µM carglumic acid, 3 µM capecitabine, and 1 µM ivermectin, none had a significant effect on the magnitude of rotifer reproduction. However, ivermectin delayed maximum reproduction from age 4–6 days and enhanced reproduction in older age classes. Lifespan was extended by exposure to 1 µM erythromycin, producing a 37% longer mean lifespan, 42% longer median lifespan, and a 33% longer maximum lifespan. In comparison, 1 µM ivermectin treatment in this experiment produced a 21% longer mean lifespan, 33% longer median lifespan, and a 22% longer maximum lifespan than control.

**Fig. 4:**
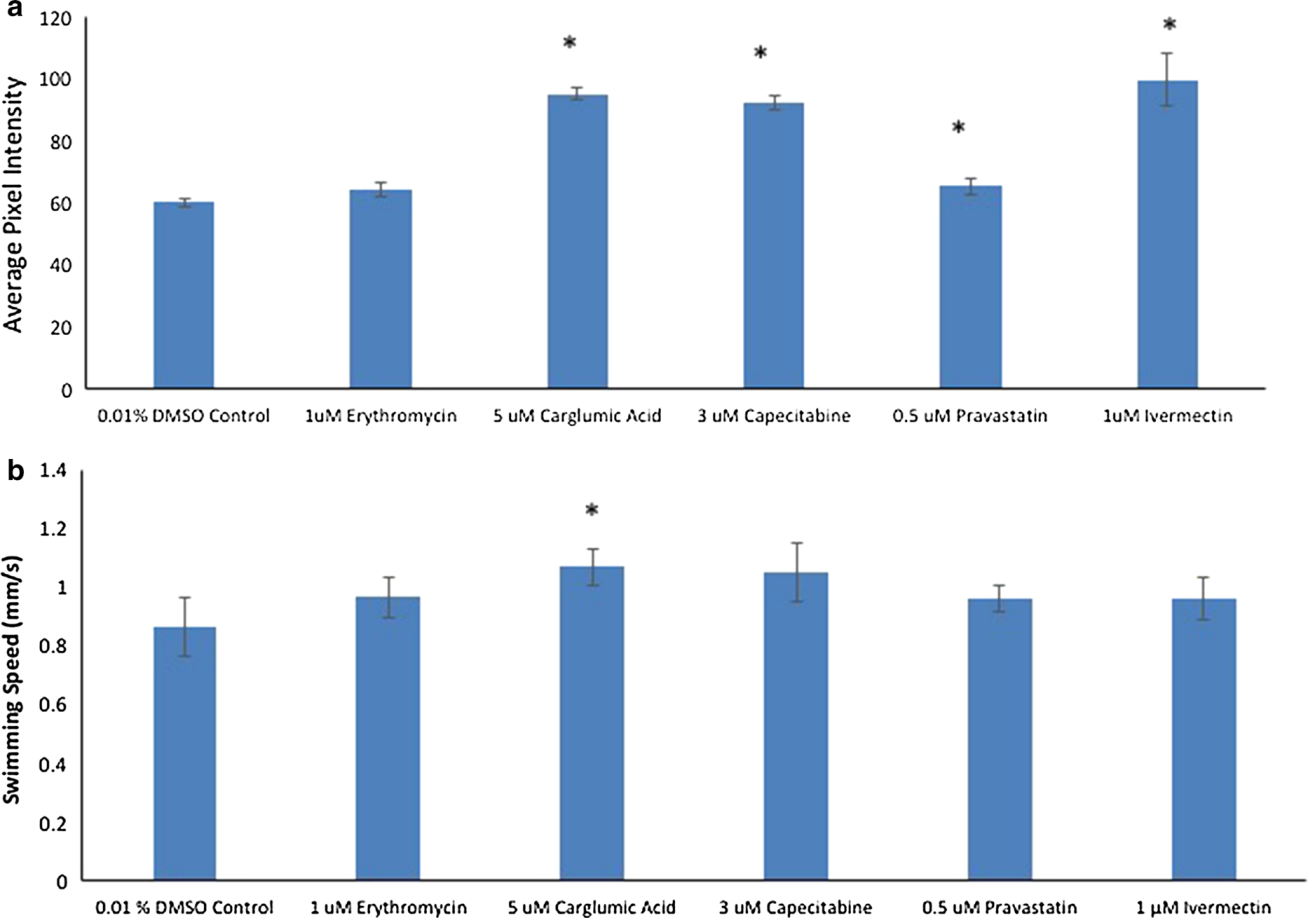
**a**, **b** Drug effects on proxies for rotifer healthspan. Mitotracker estimate of mitochondrial activity is for individual rotifers. Asterisks indicate treatments where mitochondrial activity is significantly higher than control (P < 0.05). Asterisks for swimming speed indicate that it is significantly higher than control on day 6 for 5 µM carglumic acid

In addition to lifespan extension, we also are interested in drugs capable of extending rotifer healthspan. Diminished mitochondrial activity has been associated with aging, and we used the fluorochrome Mitotracker to estimate overall mitochondrial activity (Fig. 4a). Exposure to 5 µM carglumic acid, 3 µM capecitabine, 0.5 µM pravastatin, and 1 µM ivermectin for the first 6 days of life produced significantly higher mitochondrial activity than control. Only the 1 µM erythromycin treatment failed to improve mitochondrial activity at age 6 days.

Swimming speed is another endpoint that is a useful estimate of rotifer health, and serves as a mobility proxy. *B. manjavacas* females swim continuously throughout their life, initially at an average of 0.84 mm/s as juveniles, increasing to 1.23 mm/s at age 2 days, followed by a decline back to 0.86 mm by age 4 days (Snell et al. 2016). Near death, rotifers stop swimming, fall to the bottom, and remain immobile until they die. Exposure to certain drugs might mitigate this decline in swimming speed with age. Among the drugs tested, only continuous treatment with 5 µM carglumic acid yielded significantly higher swimming speed at age 10 days than control (Fig. 4b).

We investigated whether combinations of the top candidate drugs might improve survival to older age classes more than single drug exposure. We exposed *B. manjavacas* females from birth to age 10 days to six single drugs and recorded survival. Exposure to 1 µM ivermectin, 1 µM naproxen, 1 µM erythromycin, 5 µM carglumic acid, or 0.5 µM pravastatin, all improved survival 25–39% over control. We compared this result with 15 two-way combinations of drugs, and in only two cases did we observe enhanced survival over control, and in four cases survival was considerably worse than control. However, neither of these two cases produced better survival than the single drug treatments, demonstrating the absence of additive or synergistic drug effects.

**Fig. 5:**
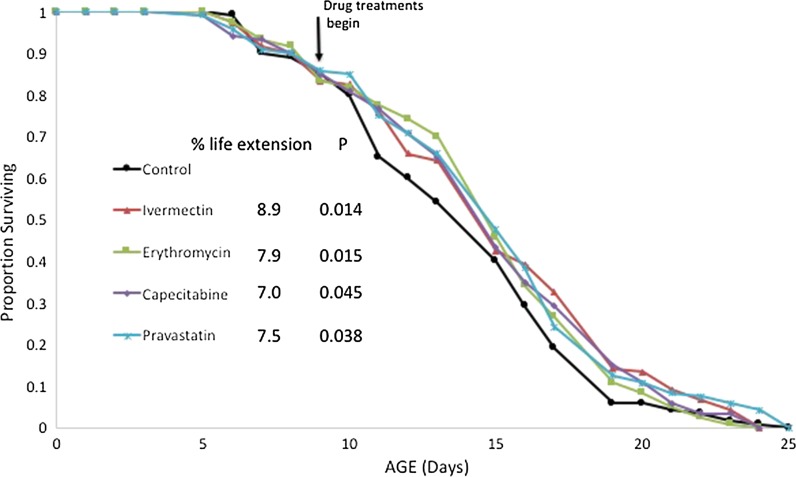
Rotifer life extension when drug treatments are initiated in middle age. Drug exposures began at age 9 days (about 55% of lifespan),  % life extension is compared to control. P values are those from a Wilcoxson test compared to control survival

There is substantial interest in finding drugs capable of slowing aging that do not require drug treatment to begin at birth. Ideally, drugs can be identified that produce significant aging benefits even when therapy is initiated in middle age. We investigated whether the five candidate drugs that we identified could produce aging benefits when therapy is started at age 9 days, the approximate midpoint of rotifer lifespan in our experimental conditions. A life table experiment was initiated where females were untreated from birth until age 9 days (Fig. 5). Then exposure to 1 µM ivermectin, 1 µM erythromycin, 3 µM capecitabine, or 0.5 µM pravastatin was initiated. Survival of all treatments was followed until the death of the last animals and the survival curves compared to control. Survival in all four drug treatments was 7–8.9% better than the control, all statistically significant at P = 0.014–0.045.

**Fig. 6:**
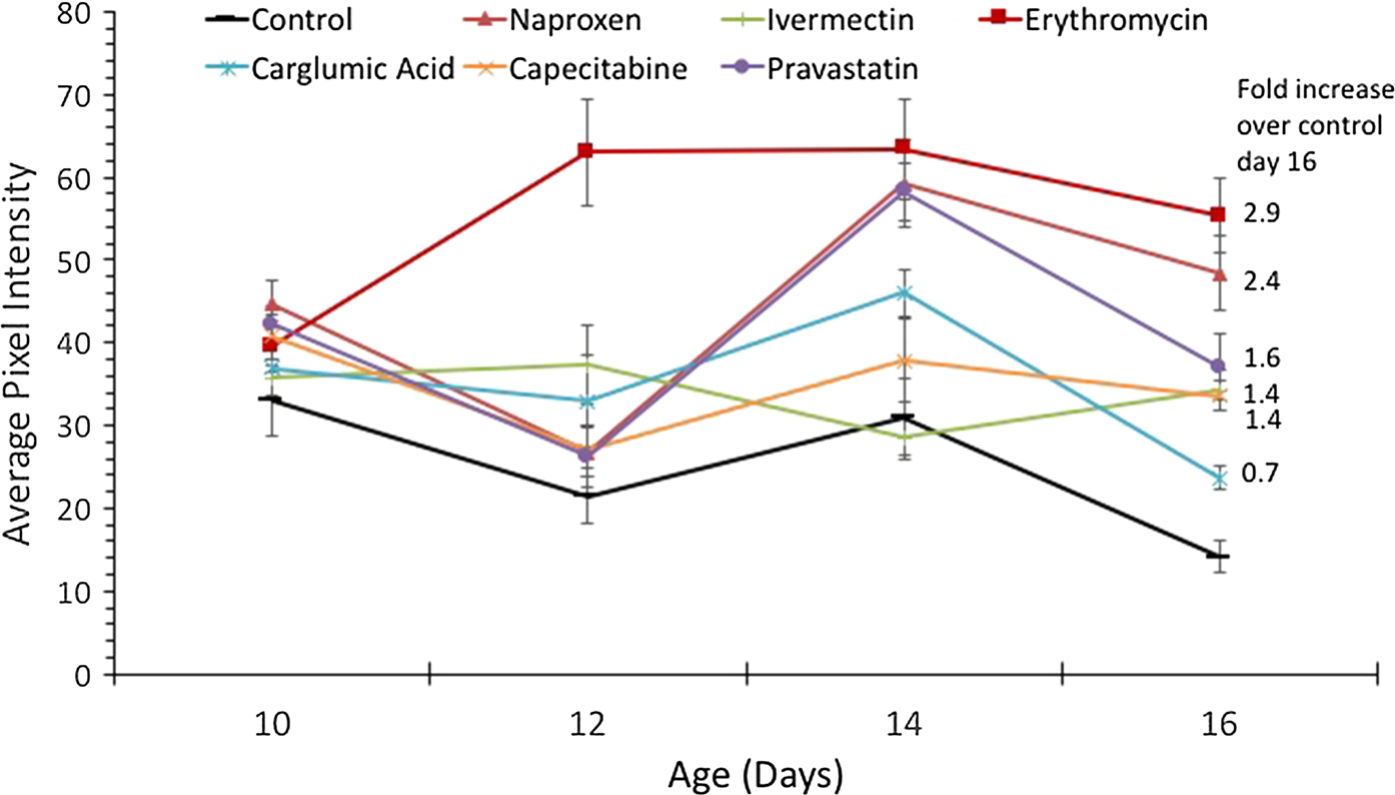
Mitotracker activity in older age classes when drug treatment is initiated in middle age. Fold increase is the amount of mitochondrial activity higher than control on day 16

Likewise, we tested the effects of drug therapy beginning in middle age on the healthspan proxies mitochondrial activity and swimming speed. When drug treatment was started on day 9 and followed through age day 16, five of the six drug treatments performed better than control. Exposure to 1 µM ivermectin, 1 µM erythromycin, 3 µM capecitabine, 1 µM naproxen, or 0.5 µM pravastatin improved mitochondrial activity over control by 1.4, 2.9, 1.4, 2.4 or 1.6-fold on day 16, respectively (Fig. 6). An ANOVA followed by Dunnett’s test yielded P < 0.0001 for all five drugs compared to control. Drug therapy beginning in midlife had less effect on preserving swimming speed in older age classes. On day 16, only the 3 µM capecitabine treatment swam significantly faster than control animals (*t* test, P = 0.018). However, on day 12, rotifer swimming speed was faster in four of the drug treatments than control (1 µM erythromycin, 3 µM capecitabine, 5 µM carglumic acid, and 0.5 µM pravastatin).

## Discussion

The significance of these results is that they demonstrate that coupling computation to experimentation can quickly identify new drug candidates with the potential to slow aging. Exploring the pool of FDA approved drugs significantly shortens drug development cycles because the safety of these compounds in humans is already established (Ashburn and Thor 2004). Most drugs bind to multiple targets (Zhou et al. 2015), so there is a strong possibility that they have undiscovered binding partners beyond their licensed targets. Thus, the pool of FDA approved drugs is likely to be rich with new targets for novel aging therapies.

The power of combining computational and experimental approaches in drug discovery using model animals has been demonstrated by Snell et al. (2016). These authors identified several drug candidates by screening three rotifer proteins for binding partners from a compound library consisting of DrugBank drugs, including 1347 FDA approved, non-nutraceutical molecules. Using survival screens, cohort life tables, analysis of swimming speed and mitochondria activity, they found three drugs, naproxen, fludarabine, and hydralazine, that extended rotifer lifespan or healthspan or both. This work was proof-of-principle of the computational model and the rotifer experimental system. This approach was expanded in the current paper where we have systematically screened proteins from most aging related genes in the GenAge database for their binding to FDA approved drugs. Identifying the top 1% of binders and removing those with high toxicity (KI > 0) using the FINDSITE^comb^algorithm (about 600 drugs), we eventually experimentally tested 31 drugs using our rotifer experimental system. From these, five drugs (ivermectin, erythromycin, capecitabine, carglumic acid, and pravastatin) demonstrated the ability to extended lifespan or healthspan or both in a variety of experiments. Only erythromycin and pravastatin have been implicated previously as drugs with aging benefits. Consequently, this work has identified promising new drug candidates and their approximate therapeutic doses for testing in vertebrate models of aging.

Another research group (Ziehm et al. 2017) has taken a similar approach, but using a different computational model to generate a short list of aging drug candidates. They identified 15 top ranked drugs each for *Drosophila melanogaster* and *Caenorhabditis elegans* that they predicted would modulate aging. However, none of the drugs on their list match our top five candidates. This is likely due to the fact that our FINDSITE^comb^ is a more general method that uses not only the PDB library, but also considers the CHEMBL library, whereas the method by Ziehm et al. is specific for *Drosophila melanogaster* and *Caenorhabditis elegans* and only focused on drug-like molecules present in PDB structures. Nevertheless, FINDSITE^comb^ also identified the same four FDA approved drugs as top ranked in the work by Ziehm et al.: DB01254(Dasatinib), DB00619(Imatinib), DB00398(Sorafenib) and DB04868(Nilotinib). However, all these kinase inhibitors were approved as cancer drugs and have serious side effects, which make them unlikely candidates for aging therapies. By comparison, one key advantage of our approach is that we applied our side-effect assessment using the killing index and eliminated these drugs from further experimental tests. However, it should be noted that Dasatinib is considered one of the first senolytic drugs and is currently being considered as a candidate for clinical trials (Kirkland and Tchkonia 2017).

Another important difference in our work is that we verified our computational predictions with in vivo animal experiments with rotifers. Ziehm et al. (2017) provided no direct experimental validation of their predictions. A further complication of using *C. elegans* for drug screening is that the worms are fed *E. coli* bacteria, which could metabolize the drugs before they affect *C. elegans*. In contrast, our experimental rotifer, *B. manjavacas,* is fed a diet of marine microalgae which are not as highly adapted to metabolize drugs as human gut bacteria like *E. coli*.

Like all invertebrates, including *Caenorhabditis elegans* and *Drosophila melanogaster*, there are limitations using rotifers as model animals to screen for drugs capable of slowing aging. Species-specific differences in drug metabolism may produce false positives or negatives for lifespan extension. Because rotifers are aquatic animals, this may cause special bioavailability problems for drug delivery. For these reasons, the drugs that we have identified as producing lifespan and healthspan extension in this study should be regarded as a working hypothesis until confirmed in a mammalian model.

An advantage of using FDA approved drugs is that their mechanisms of action are usually known. For example, ivermectin is highly efficacious against a variety of parasitic infections in animals (Laing et al. 2017). It is known for blocking of glutamate-gated chloride channels in parasitic nematodes, inhibiting motility, feeding and reproduction. Although this is ivermectin’s licensed application, at micromolar concentrations it is known to bind to a wider range of ligand-gated channels, including GABA, glycine, histamine, and nicotinic acetylcholinesterase receptors (Wolstenholme and Rogers 2005). In mammals, ivermectin has been shown to bind to the ligand binding domain of the farnesoid X receptor in mice, decreasing serum glucose and cholesterol levels (Jin et al. 2013). Ivermectin also inhibits proliferation and induces apoptosis in several human cancer cells (Melotti et al. 2014). Overdoses of ivermectin in humans cause cardiotoxicity, neurotoxicity, and adverse effects in the gastrointestinal tract (Yang 2012). Because of this promiscuity in binding partners, it is perhaps not surprising that ivermectin also affects metabolic pathways modulating aging in rotifers.

Erythromycin is another drug with interesting effects on rotifer lifespan and healthspan. It is a 14-membered ring macrolide used to treat chronic inflammatory diseases. In addition, erythromycin has been shown to slow aging in yeast (Holbrook and Menninger 2002). The *Saccharomyces cerevisiae* strain K65-3D grown in 16 g/mL erythromycin had a mean life span that was 27% longer than untreated yeast cells. Although this result was intriguing, there have been no follow-up studies and no demonstration of similar effects in animals until our work with rotifers reported a 37% increase in mean lifespan.

Snell et al. (2016) reported that rotifers treated with 1 µM naproxen had a 14% longer mean lifespan than controls. Naproxen is a nonsteroidal anti-inflammatory drug (NSAID) for relieving pain, fever, swelling, and stiffness that is a nonselective COX inhibitor. These authors hypothesized that naproxen effects were manifested through an anti-inflammatory mechanism.

The cancer drug capecitabine is used in chemotherapy to treat breast, gastric and colorectal cancer. Capecitabine is metabolised to 5-fluoro-2-deoxyuridine (5-FU), which in turn is a thymidylate synthetase inhibitor (Shimma et al. 2000). Inhibition of this enzyme reduces the synthesis of thymidine monophosphate, which is the active form of thymidine required for the de novo synthesis of DNA. The drug 5-FU has been used extensively in rotifer life table experiments to inhibit hatching of eggs, which eliminates the necessity of removing offspring from maternal females. Offspring removal considerably increases the effort required to perform rotifer life table experiments. In describing the use of 5-FU, Snell et al. (2012) reported a consistent 20% extension of mean lifespan in their experiments, but were unable to provide an explanation. We did not observe such a lifespan extension for capecitabine in our life table experiments, but it yielded lifespan benefits in primary and secondary survival screens and improved mitochondrial function in older age classes.

Pravastatin acts as a lipoprotein-lowering drug by binding to the active site and inhibiting the function of the enzyme hydroxymethylglutaryl-CoA (HMG-CoA) reductase. This helps prevent age-related cardiovascular disease. A combination of statins, including pravastatin, inhibited both farnesylation and geranylgeranylation of progerin and prelamin A in a mouse model of premature aging (Varela et al. 2008). This markedly slowed aging by retarding growth, weight loss, hair loss and bone defects, resulting in substantial lifespan extension. The drug carglumic acid is used to treat hyperammonanemia in patients with N-acetylglutamate synthase deficiency, but has no reported effect on aging processes.

One of the most important results of this work is identifying drugs that heretofore have not been implicated as candidates for aging therapy. As exciting as this prospect is, perhaps more promising is the observation that drug therapy can be initiated in midlife and still produce aging benefits. A drug may produce highly desirable aging benefits, but if therapy needs to be initiated at birth and continued throughout the lifespan, few people are going to comply with this therapeutic regime. However, if therapy can begin at midlife, patients are much more likely to comply. Four of the drugs that we tested, ivermectin, erythromycin, capecitabine, and pravastatin, all produced significant lifespan extension (7–8.9%) when started at age 9 days, about midway through the average rotifer lifespan. These same drugs plus naproxen also improved mitochondrial function in older age classes, 1.4–2.9-fold over control. In addition, midlife treatment with erythromycin, capecitabine, carglumic acid, and pravastatin improved swimming performance in some older age classes, compared to control. Together these results are quite encouraging because they demonstrate that proper doses of particular drugs can provide lifespan and healthspan benefits to animals, even if therapy begins in midlife.

Our results have identified drugs that are strong candidates for aging therapy and demonstrated their efficacy in an in vivo rotifer model. The next step in the development of these compounds for human therapy is to test them in mammals, explore a range of therapeutic doses, and the optimal timing of drug delivery. If these trials are successful, this should provide a strong indication of their likely success in human patients.
